# Supplementary material for: Rewiring of the promoter-enhancer interactome and regulatory landscape in glioblastoma orchestrates gene expression underlying neurogliomal synaptic communication
Source: Nat Commun. 2023 Oct 13;14:6446. doi: 10.1038/s41467-023-41919-x (PMC10576091; doi:10.1038/s41467-023-41919-x)
Supplement: Supplementary file 3 — Description of Additional Supplementary Files [file 41467_2023_41919_MOESM3_ESM.pdf]

Description of Additional Supplementary Files

File Name: Supplementary Data 1
Description: : Differential expression analysis of the 15 patient-derived GB lines *versus* normal astrocytes (two-tailed Wald's likelihood test with BenjaminiHochberg correction).

File Name: Supplementary Data 2
Description: Differential expression analysis of the 15 patient-derived GB lines *versus* OPCs (two-tailed Wald's likelihood test with Benjamini-Hochberg correction).

File Name: Supplementary Data 3
Description: Summary of Next Generation Sequencing data including ChIPseq, ATAC-seq, HiChIP and CUT&RUN.

File Name: Supplementary Data 4
Description: List of oligonucleotides used for ATAC-seq library preparation

File Name: Supplementary Data 5
Description: List of qPCR primers.
