# Supplementary material for: Rewiring of the promoter-enhancer interactome and regulatory landscape in glioblastoma orchestrates gene expression underlying neurogliomal synaptic communication
Source: Nat Commun. 2023 Oct 13;14:6446. doi: 10.1038/s41467-023-41919-x (PMC10576091; doi:10.1038/s41467-023-41919-x)
Supplement: Supplementary file 1 — Supplementary Information [file 41467_2023_41919_MOESM1_ESM.pdf]

Supplementary Information

Rewiring of the promoter-enhancer interactome and regulatory landscape in glioblastoma orchestrates gene expression underlying neurogliomal synaptic communication

Chaitali Chakraborty[1,2,3], Itzel Nissen[1,2,3], Craig A. Vincent[1,2], Anna-Carin Hägglund[1,2], Andreas Hörnblad[1] and Silvia Remeseiro[1,2,*]

[1] Umeå Centre for Molecular Medicine (UCMM), Umeå University, Umeå, Sweden.

[2] Wallenberg Centre for Molecular Medicine (WCMM), Umeå University, Umeå, Sweden.

[3] These authors contributed equally: CC and IN

* Correspondence should be addressed to S.R.: silvia.remeseiro@umu.se

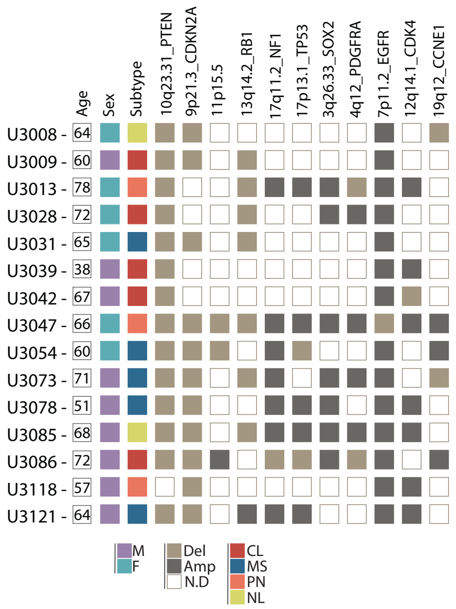

Supplementary Figure 1: Overview of the 15 patient-derived GB lines in this study. Panel displaying information about the 15 GB lines in this study with regards to age, sex (M: male, F: female), GB subtype (CL: classical, MS: mesenchymal, PN: proneural, N: neural), and different chromosomal alterations (Del: deletion, Amp: amplification, N.D: not determined).

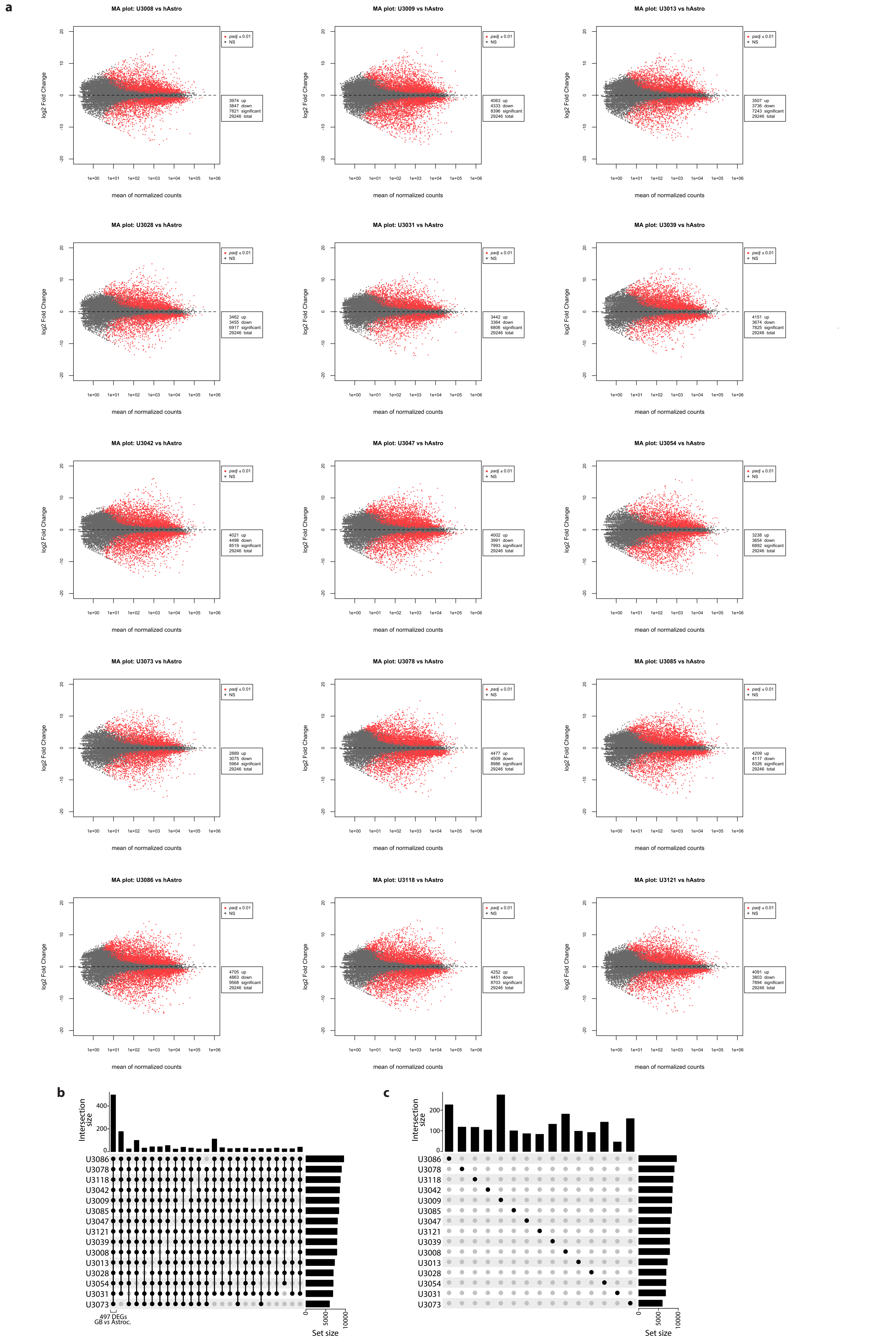

Supplementary Figure 2: Differential expression analysis of GB *vs* normal astrocytes. a) MA plots showing genes differentially expressed (DEGs, red dots) in each of the 15 GB lines *vs* normal astrocytes. b,c) UpSet plots showing the intersections of the DEGs from each of the 15 pairwise comparisons displaying genes differentially expressed in ≥10 GB lines with ≥20 genes (b) or displaying the DEGs unique to each GB line (c).

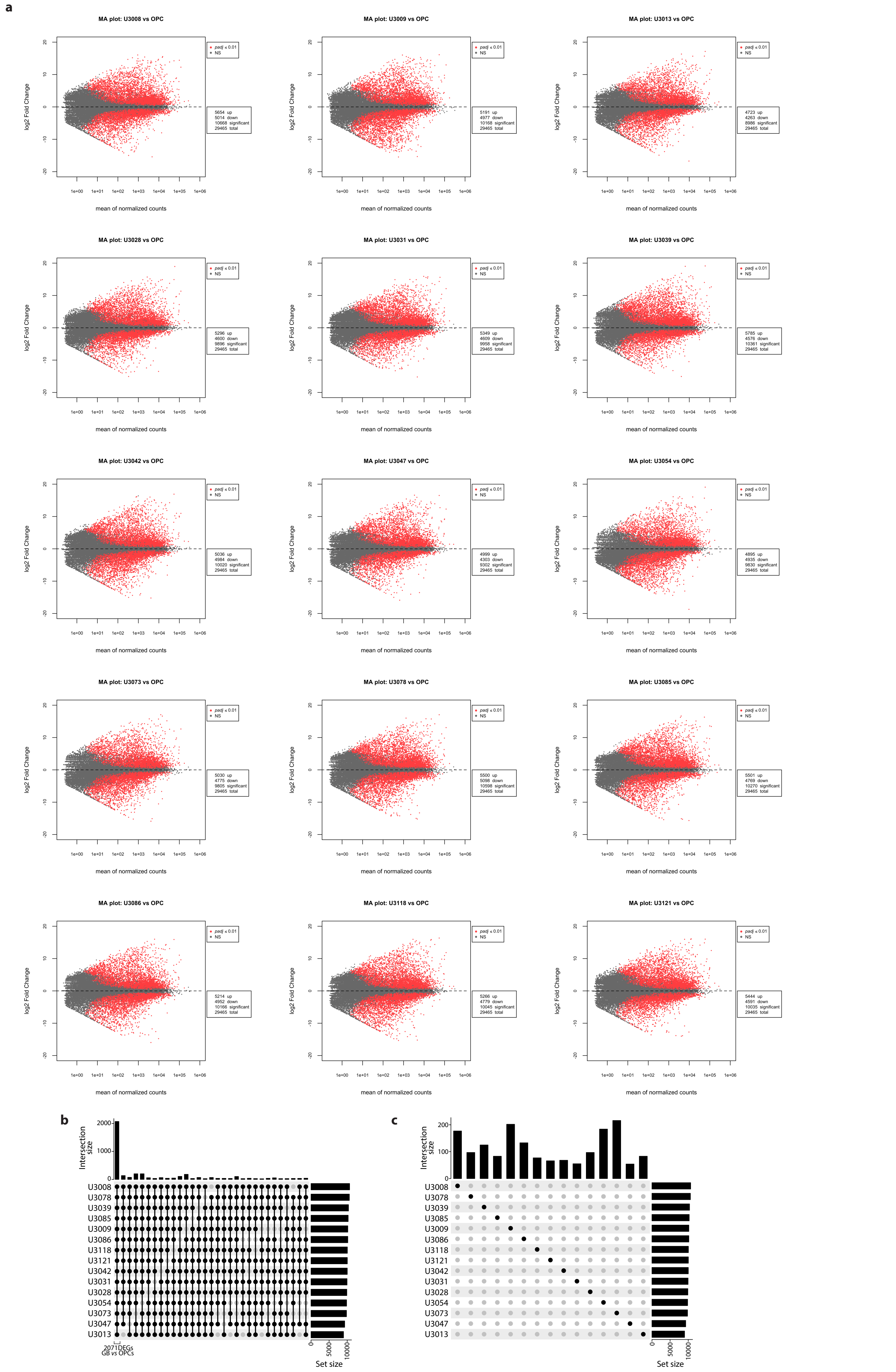

Supplementary Figure 3: Differential expression analysis of GB *vs* OPCs. a) MA plots showing genes differentially expressed (DEGs, red dots) in each of the 15 GB lines *vs* OPCs. b,c) UpSet plots showing the intersections of the DEGs from each of the 15 pairwise comparisons displaying genes differentially expressed in ≥10 GB lines with ≥20 genes (b) or displaying the DEGs unique to each GB line (c).

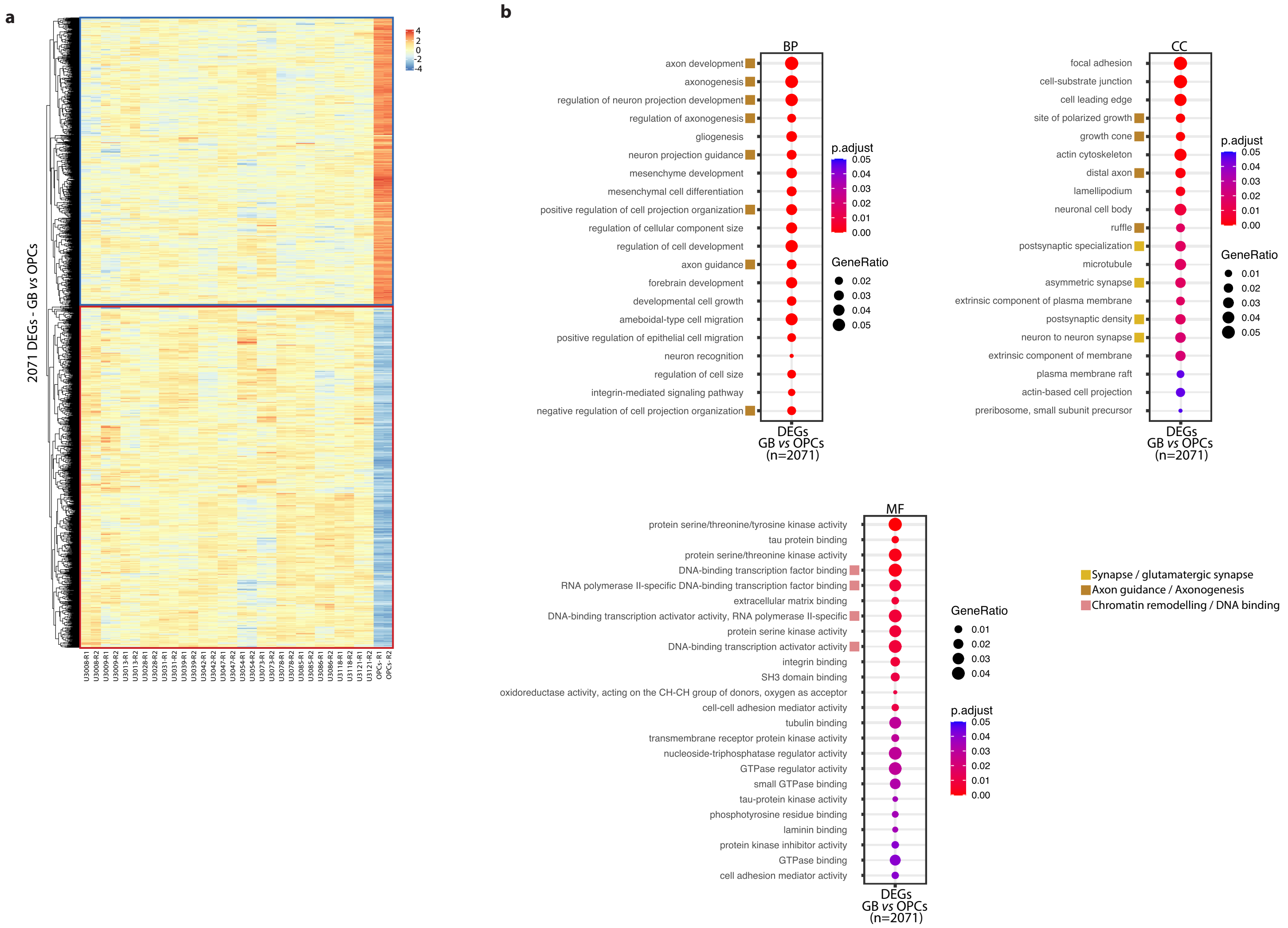

Supplementary Figure 4: Gene expression changes in GB in comparison to OPCs. a) Expression of the 2071 DEGs in all 15 GB lines *vs* OPCs determined by RNA-seq (rlog normalized counts). b) Top Gene Ontology terms enriched in the 2071 DEGs GB *vs* OPCs (p-value: hypergeometric test with Benjamini correction).

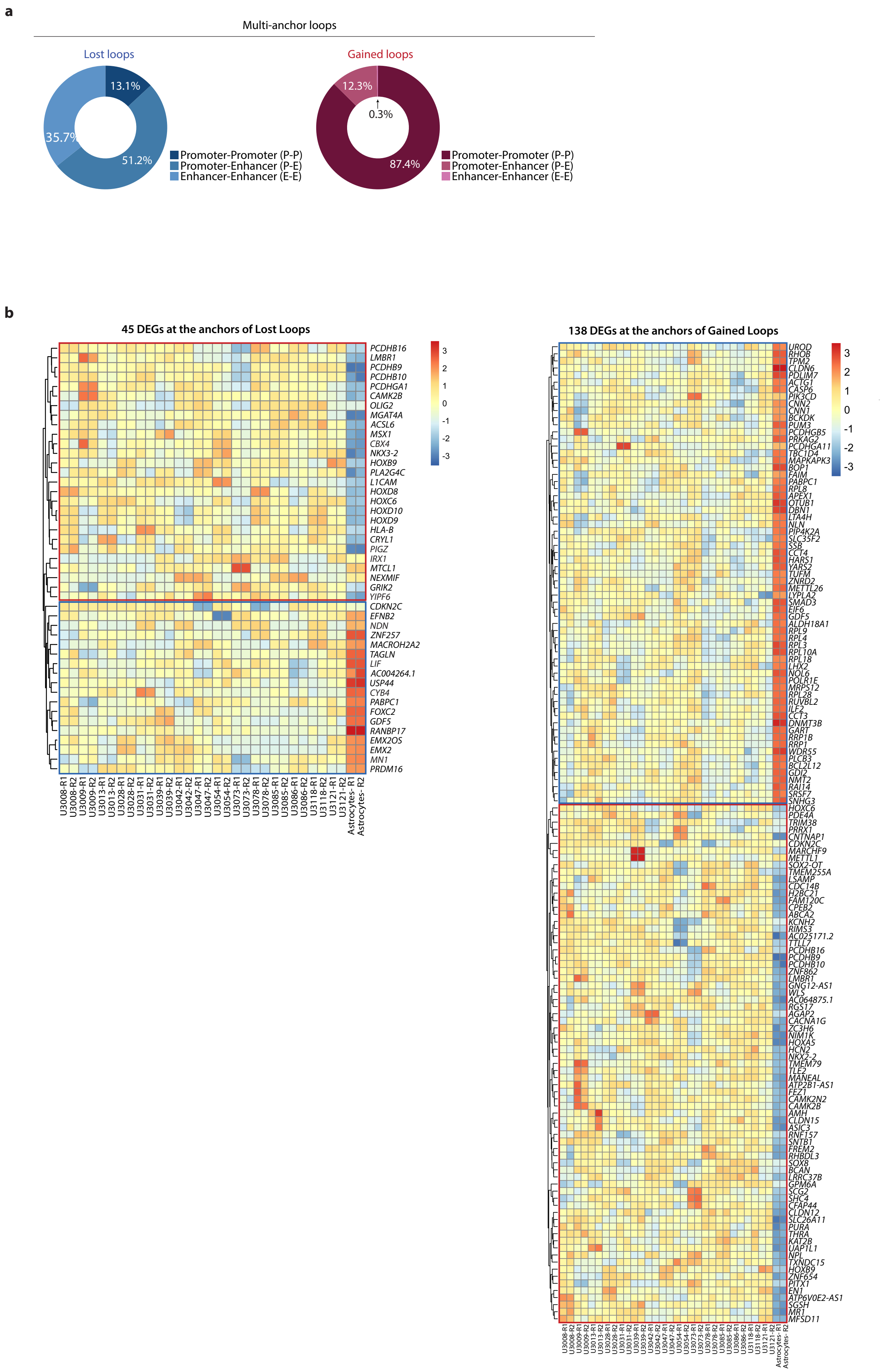

Supplementary Figure 5: Changes in the expression of genes located at differential loops. a) Annotation of lost and gained multi-anchor loops as promoter-promoter, promoter-enhancer or enhancer-enhancer loops. b) Expression of 45 DEGs located at the anchors of lost loops (left) and 138 DEGs at the anchors of gained loops (right), in all 15 GB lines and normal astrocytes as determined by RNA-seq (rlog normalized counts). Source Data are provided as a Source Data file for Supplementary Fig. 5a.

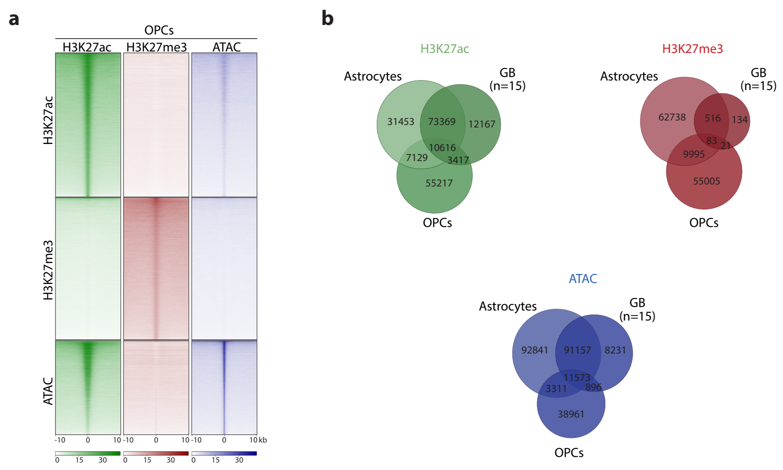

Supplementary Figure 6: Mapping of histone marks and chromatin accessible regions in OPCs. a) Read distribution of histone marks and ATAC signal around the indicated ±10kb regions in OPCs. b) Overlap of histone marks and ATAC regions in astrocytes, OPCs and GB lines.

a

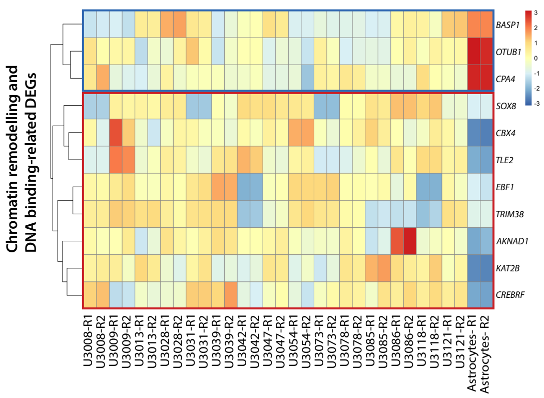

b

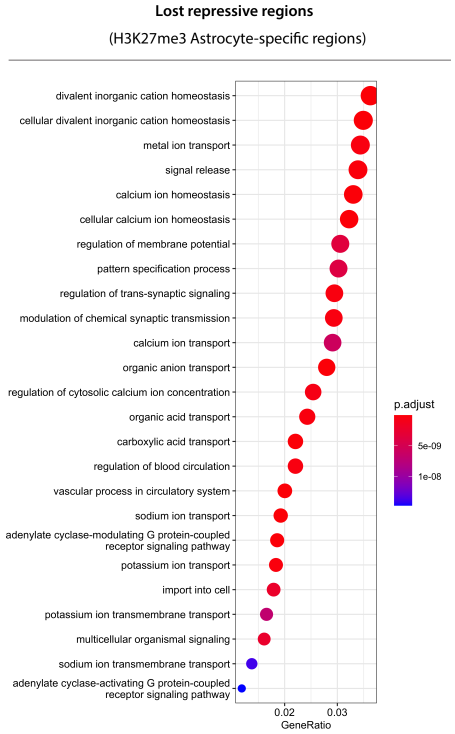

c

Proximal (<2kb TSS) gained regions (GB-specific):

			H3K27ac		ATAC		H3K4me3	
**TF**	**Motif**	**Sequence**	**Motif Logo**	**p-value**	**Motif Logo**	**p-value**	**Motif Logo**	**p-value**
En1	En1(Homeobox)/SUM149-EN1-ChIP-Seq(GSE120957)/Homer	NDCTAATTAS		1.00E-56		1.00E-34		1.00E-43
GSC	GSC(Homeobox)/FrogEmbryos-GSC-ChIP-Seq(DRA000576)/Homer	RGGATTAR		1.00E-19		1.00E-16		1.00E-52
HOXB13	HOXB13(Homeobox)/ProstateTumor-HOXB13-ChIP-Seq(GSE56288)/Homer	TTTTATKRGG		1.00E-32		1.00E-16		1.00E-31
HOXD10	Hoxd10(Homeobox)/ChickenMSG-Hoxd10.Flag-ChIP-Seq(GSE86088)/Homer	GGCMATGAAA		1.00E-18		1.00E-14		1.00E-14
LHX2	Lhx2(Homeobox)/HFSC-Lhx2-ChIP-Seq(GSE48068)/Homer	TAATTAGN		1.00E-25		1.00E-39		1.00E-31
PITX1	Pitx1(Homeobox)/Chicken-Pitx1-ChIP-Seq(GSE38910)/Homer	TAATCCCN		1.00E-12		1.00E-08		1.00E-10
SIX1	Six1(Homeobox)/Myoblast-Six1-ChIP-Chip(GSE20150)/Homer	GKVTCADRTTWC		1.00E-07		1.00E-02	-	-
SMAD3	Smad3(MAD)/NPC-Smad3-ChIP-Seq(GSE36673)/Homer	TWGTCTGV		1.00E-07		1.00E-09	-	-

d

Distal (>2kb TSS) gained regions (GB-specific):

			H3K27ac		ATAC		H3K4me3	
**TF**	**Motif**	**Sequence**	**Motif Logo**	**p-value**	**Motif Logo**	**p-value**	**Motif Logo**	**p-value**
En1	En1(Homeobox)/SUM149-EN1-ChIP-Seq(GSE120957)/Homer	NDCTAATTAS		1.00E-12		1.00E-14		1.00E-11
GSC	GSC(Homeobox)/FrogEmbryos-GSC-ChIP-Seq(DRA000576)/Homer	RGGATTAR		1.00E-20		1.00E-08		1.00E-09
HOXB13	HOXB13(Homeobox)/ProstateTumor-HOXB13-ChIP-Seq(GSE56288)/Homer	TTTTATKRGG		1.00E-12		1.00E-20		1.00E-25
HOXD10	Hoxd10(Homeobox)/ChickenMSG-Hoxd10.Flag-ChIP-Seq(GSE86088)/Homer	GGCMATGAAA		1.00E-08		1.00E-32		1.00E-16
LHX2	Lhx2(Homeobox)/HFSC-Lhx2-ChIP-Seq(GSE48068)/Homer	TAATTAGN		1.00E-19		1.00E-34		1.00E-17
PITX1	Pitx1:Ebox(Homeobox,bHLH)/Hindlimb-Pitx1-ChIP-Seq(GSE41591)/Homer	YTAATTRAWWCCAGATGT		1.00E-23	-	-		1.00E-04
SIX1	Six1(Homeobox)/Myoblast-Six1-ChIP-Chip(GSE20150)/Homer	GKVTCADRTTWC		1.00E-03		1.00E-15		1.00E-05
SMAD3	Smad3(MAD)/NPC-Smad3-ChIP-Seq(GSE36673)/Homer	TWGTCTGV		1.00E-23		1.00E-17		1.00E-05
RORa	RORa(NR)/Liver-Rora-ChIP-Seq(GSE101115)/Homer	AAWCTAGGTCARDNN	-	-		1.00E-04	-	-
EBF1	EBF1(EBF)/Near-E2A-ChIP-Seq(GSE21512)/Homer	GTCCCCWGGGGA		1.00E-12		1.00E-04	-	-
THRB	THRb(NR)/Liver-NR1A2-ChIP-Seq(GSE52613)/Homer	TRAGGTCA		1.00E-06		1.00E-02	-	-

e

HiChIP (anchors of differential loops)

**TF**	**Motif**	**Sequence**	**Motif Logo**	**p-value**
PITX1	Pitx1(Homeobox)/Chicken-Pitx1-ChIP-Seq(GSE38910)/Homer	TAATCCCN		1.00E-12
SMAD3	Smad3(MAD)/NPC-Smad3-ChIP-Seq(GSE36673)/Homer	TWGTCTGV		1.00E-20

Supplementary Figure 7: GO terms and TF motifs around which histone marks and chromatin accessible regions are redistributed in GB. a) Expression of DEGs related to chromatin remodelling and DNA binding located <2kb from a gained active/open peak. b) Top 25 GO terms enriched for the genes proximal to the lost repressive regions in GB (peaks <2kb TSS) (p-value: hypergeometric test with Benjamini correction). c,d) Summary of the TF binding motifs enriched at proximal (<2kb TSS) (c) or distal (>2kb TSS) (d) gained regions in GB for H3K27ac, ATAC and H3K4me3, including motif, sequence and p-values: hypergeometric test. e) Summary of the TF binding motifs enriched at the anchors of the differential loops, including motif, sequence and p-values: hypergeometric test.

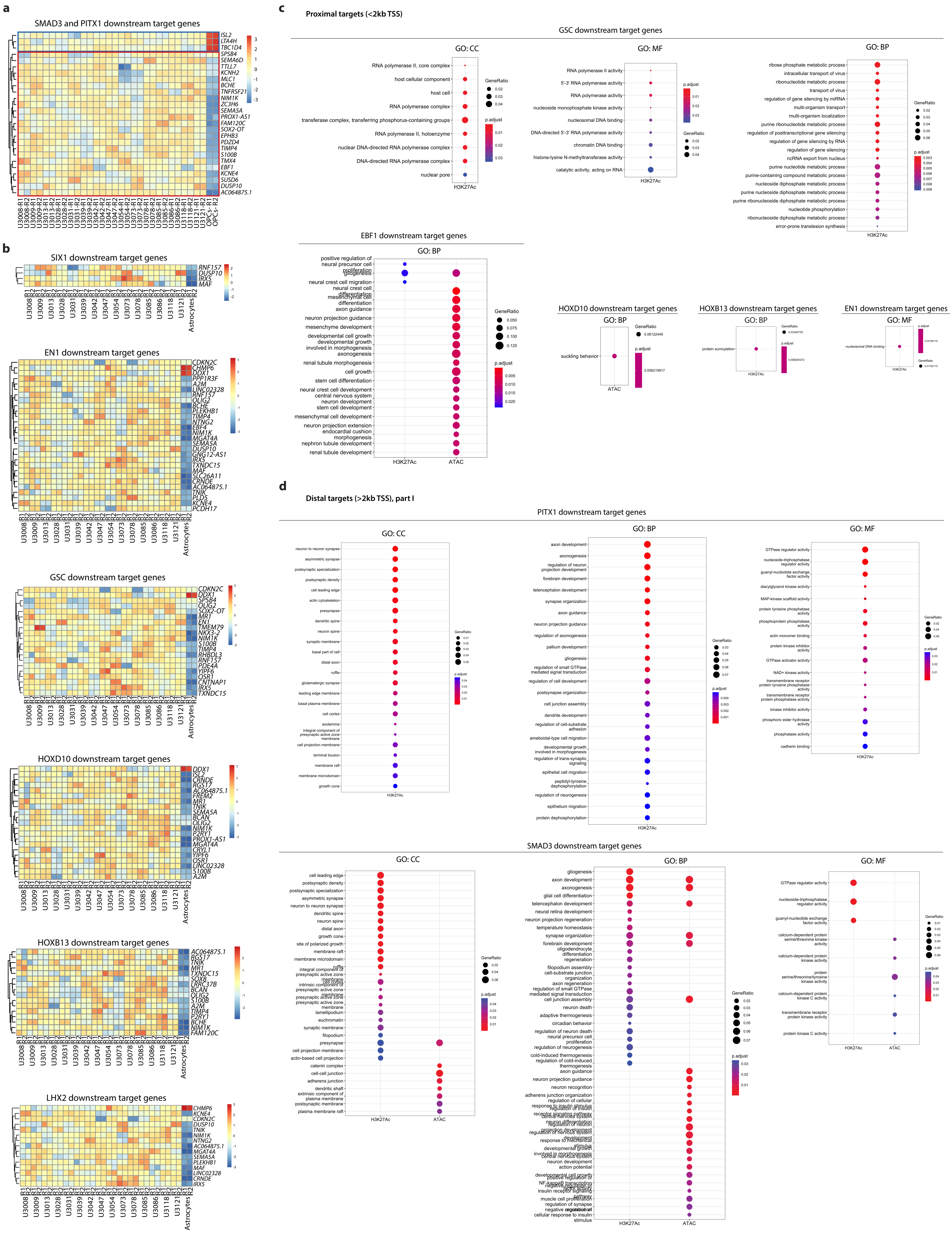

Supplementary Figure 8: Expression and GO term enrichment for TFs downstream target genes. a) Expression of SMAD3 and PITX1 downstream targets that belong to the 2071 DEGs in OPCs and 15 GB lines. b) Expression of the SIX1, EN1, GSC, HOXD10, HOXB13 and LHX2 downstream target genes (i.e., motif enrichment at a differential peak <2kb TSS) that are DEGs in GB *vs* normal astrocytes. c) GO terms enriched for the GSC, EBF1, HOXD10, EN1 and HOXB13 proximal downstream target genes (<2kb TSS) (p-value: hypergeometric test with Benjamini correction). d) GO terms enriched for the PITX1 and SMAD3 distal downstream target genes (>2kb TSS) (p-value: hypergeometric test with Benjamini correction).

Distal targets (>2kb TSS), part II

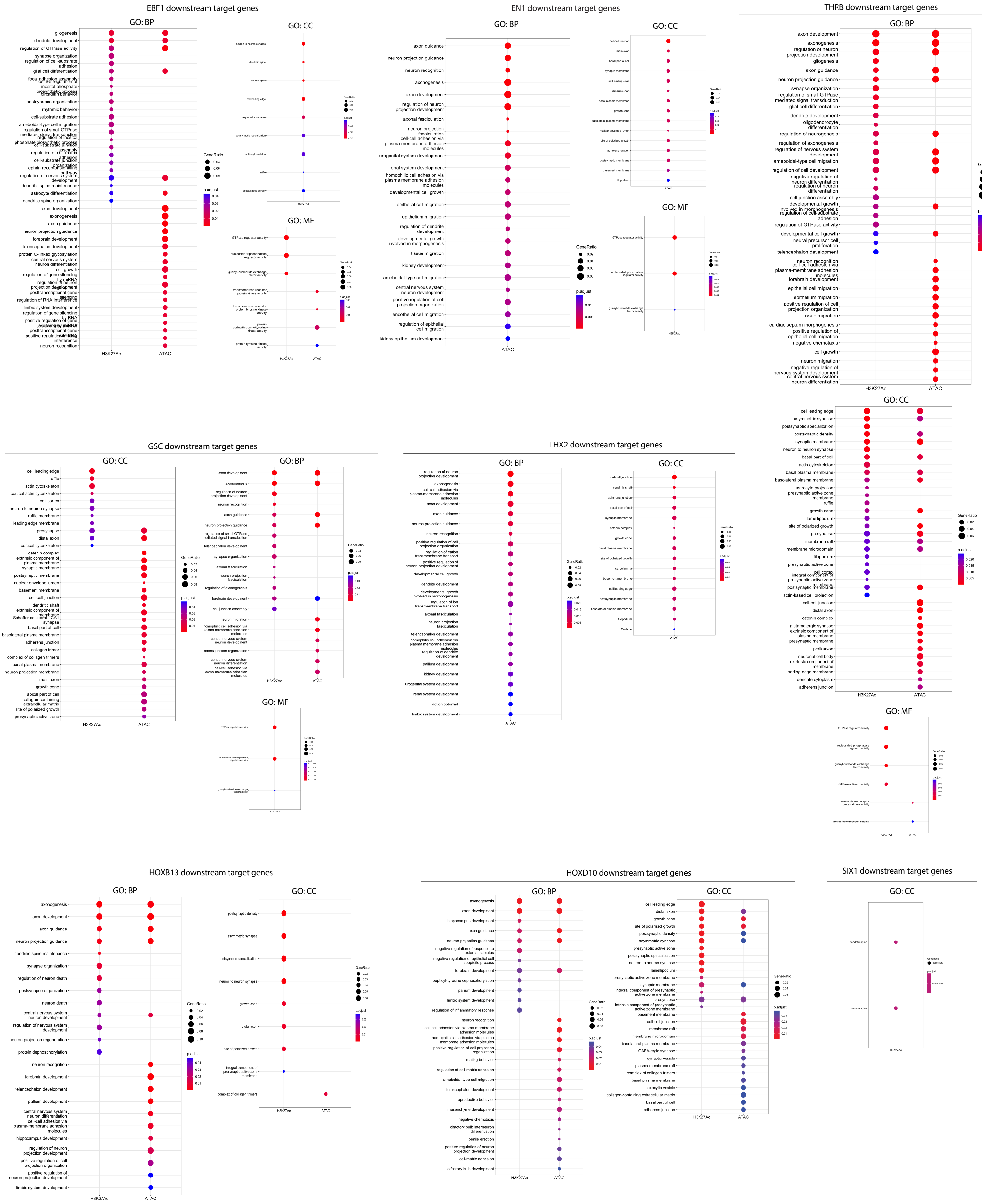

Supplementary Figure 9: GO term enrichment for TFs downstream target genes. GO terms enriched for the EBF1, EN1, THRB, GSC, LHX2, HOXB13, HOXD10 and SIX1 distal downstream target genes (>2kb TSS) (p-value: hypergeometric test with Benjamini correction).

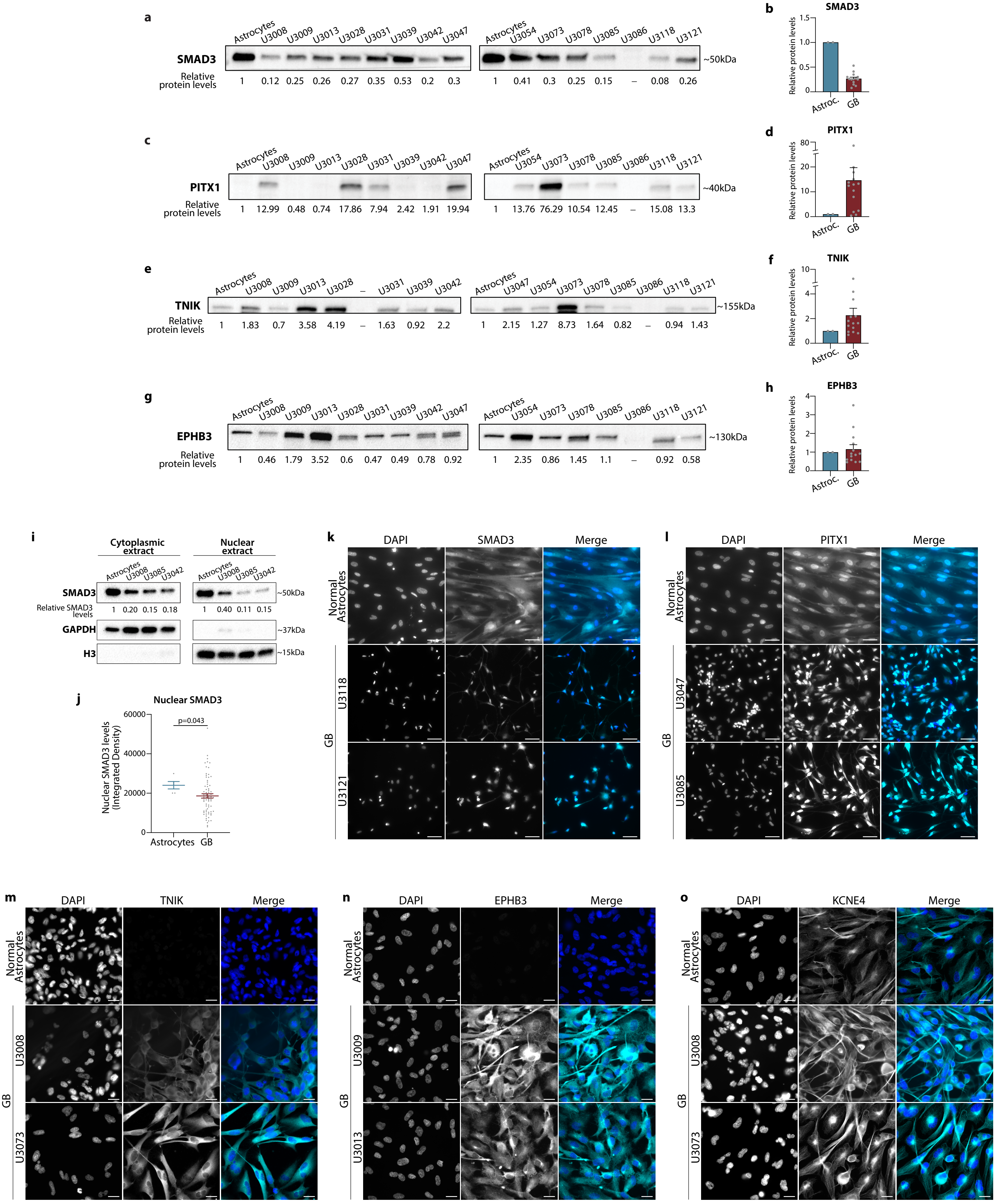

Supplementary Figure 10: Protein levels for SMAD3, PITX1 and some of their downstream targets in astrocytes and 15 GB lines. a-h) Protein levels for SMAD3 (a,b), PITX1 (c ,d), TNIK (e ,f) and EPHB3 (g, h) assessed by western-blot using the corresponding specific antibodies (a, c, e, g) in normal astrocytes and 15 GB lines (one experiment). Protein levels relative to astrocytes were determined by densitometric analysis (b, d, f, h). i) SMAD3 levels in cytoplasmic and nuclear extracts in 3 GB patient-derived lines relative to normal astrocytes, as assessed by densitometric analysis (one experiment). j) Nuclear SMAD3 levels in 15 GB lines and normal astrocytes as determined by quantification of SMAD3 nuclear signal from immunofluorescence staining (mean ± SEM, two-sided Kolmogorov-Smirnov test; GB (n=15 lines) and astrocytes (n=1 line), n=5 fields imaged per line). k-o) Immunofluorescence staining of SMAD3 (k), PITX1 (l), TNIK (m), EPHB3 (n) and KCNE4 (o) in astrocytes and GB lines (2 representative GB lines displayed). Scale bars: 50 μm (k, l) and 25 μm (m-o). Source Data are provided as a Source Data file for Supplementary Fig. 10j.

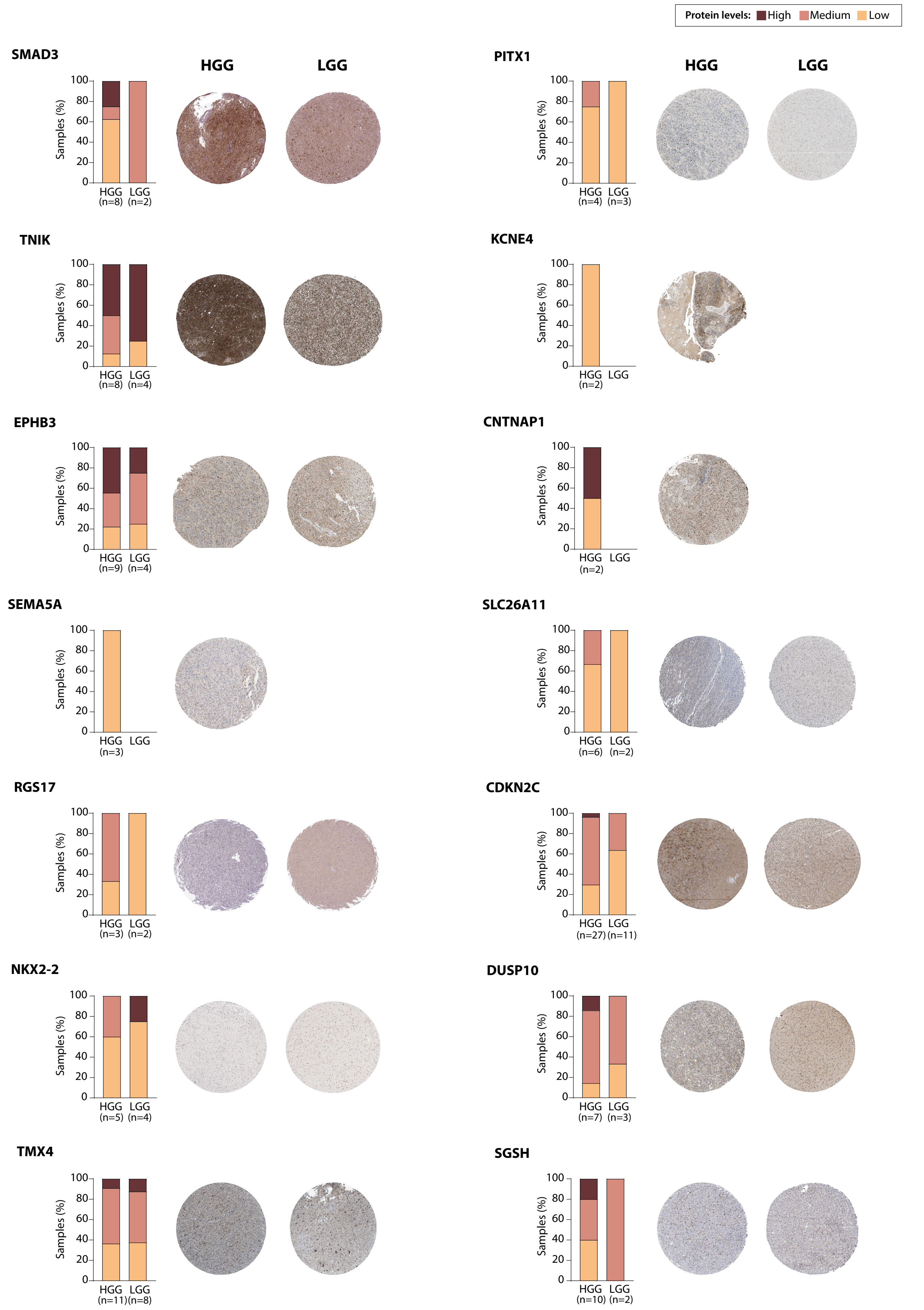

Supplementary Figure 11: Protein levels for SMAD3, PITX1 and some of their downstream targets in tumour sections from high (HGG) and low-grade (LGG) gliomas. Protein levels retrieved from the Human Protein Atlas (HPA) represented as percentage of samples with high, medium or low protein expression levels in tissue sections from High-Grade Glioma (HGG) and Low-Grade Glioma (LGG) patients (for SMAD3 n=8 HGG, n=2 LGG; PITX1 n=4 HGG, n=3 LGG; TNIK n=8 HGG, n=4 LGG; KCNE4 n=2 HGG; EPHB3 n=9 HGG, n=4 LGG; CNTNAP1 n=2 HGG; SEMA5A n=3 HGG; SLC26A11 n=6 HGG, n=2 LGG; RGS17 n=3 HGG, n=2 LGG; CDKN2C n=27 HGG, n=11 LGG; NKX2-2 n=5 HGG, n=4 LGG; DUSP10 n=7 HGG, n=3 LGG; TMX4 n=11 HGG, n=8 LGG; SGSH n=10 HGG, n=2 LGG). Source Data are provided as a Source Data file for Supplementary Fig. 11.

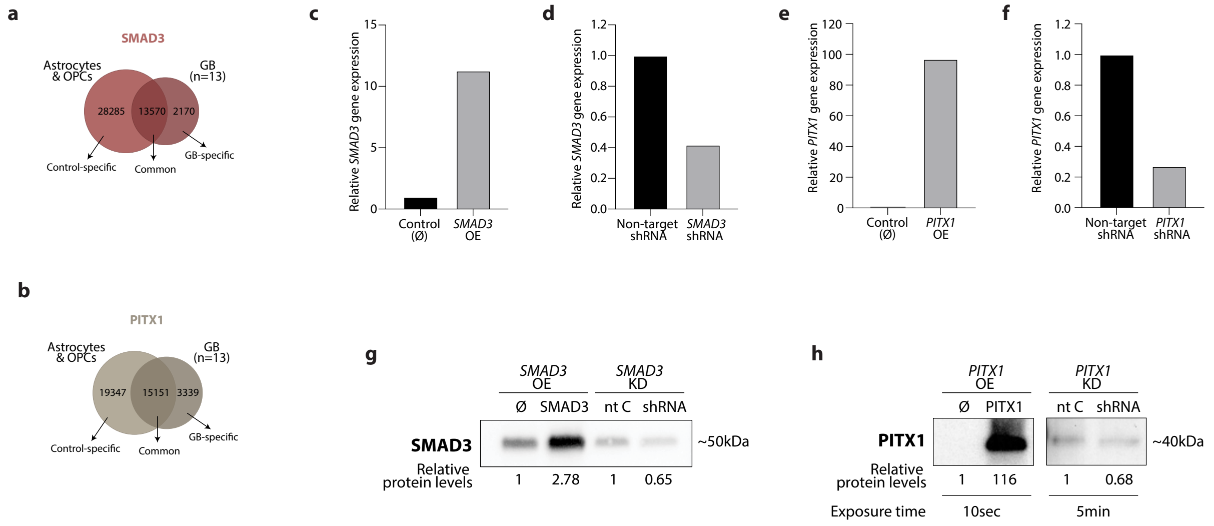

Supplementary Figure 12: Gene expression and protein levels in *SMAD3* and *PITX1* overexpression and knockdown stable lines. a,b) Overlap of SMAD3 (a) and PITX1 (b) binding sites in 13 GB lines *versus* the control astrocytes and OPCs. c,d) *SMAD3* gene expression levels determined upon *SMAD3* overexpression (OE) (c) or knockdown (KD) (d) relative to the corresponding control lines (mean, 3 qPCR technical replicates per condition, one experiment). e,f) *PITX1* gene expression levels determined upon *PITX1* overexpression (OE) (e) or knockdown (KD) (f) relative to the corresponding control lines (mean, 3 independent qPCR replicates per condition, one experiment). g,h) Protein levels for SMAD3 (g) and PITX1 (h) assessed by immunoblotting in the OE and KD lines alongside the corresponding controls (one experiment; Control ∅: empty vector control; nt C: non-target shRNA control). Source Data are provided as a Source Data file for Supplementary Fig. 12c-f.

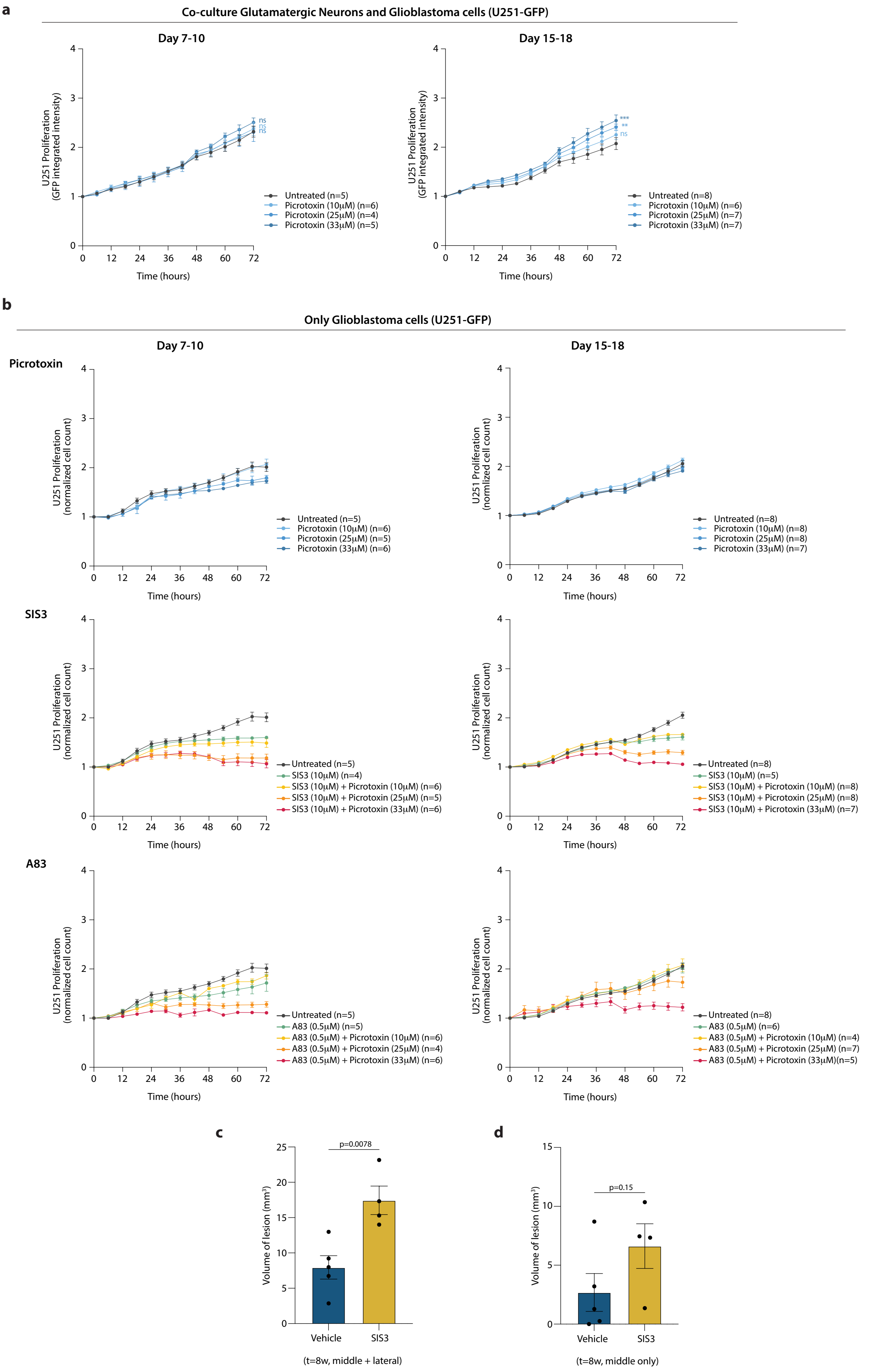

Supplementary Figure 13: Proliferation of GB cells and tumour growth upon SMAD3 inhibition. a) Proliferation curves depicting the growth of U251 cells (measured as normalized GFP integrated intensity) in co-culture with glutamatergic neurons in the 72h after seeding at day 6 (left) or day 14 (right) at different concentrations of picrotoxin (mean ± SEM, multiple unpaired two-sided t-test, t=72h, *p<0.01, **p<0.005, ***p<0.000005, exact p-values in Source Data file) [(a, left) n=5 in U, P33, n=6 in P10 and n=4 in P25; (a, right) n=8 in U, n=6 in P10 and n=7 in P25, P33; where U and P denote untreated and picrotoxin, respectively, and the numbers indicate μM concentration]. b) Proliferation curves of U251 cells in the absence of neurons (measured as normalized cell count) in the 72h after seeding at day 6 (left) or day 14 (right) and under the same drug treatments as used in Figure 7e,g. [(b, P, left) n=5 in U, P25 and n=6 in P10, P33; (b, P, right) n=8 in U, P10, P25 and n=7 in P33; (b, S, left) n=5 in U, S10+P25, n= 4 in S10 and n=6 in S10+P10, S10+P33; (b, S, right) n=8 in U, S10+P10, S10+P25, n=5 in S10 and n=7 in S10+P33; (b, A, left) n=5 in U, A0.5, n=6 in A0.5+P10, A0.5+P33 and n=4 in A0.5+P25; (b, A, right) n=8 in U, n=6 in A0.5, n= 4 in A0.5+P10, n=7 in A0.5+P25 and n=5 in A0.5+P33; where U, P, S and A denote untreated, picrotoxin, SIS3 and A83, respectively, and the numbers indicate μM concentration]. c,d) Volume of pathological lesions in vehicle- or SIS3-treated mice at t=8 weeks after tumour induction (n=5 and n=4, respectively) measured as the tumour volume in the medial part of the brain plus the volume of the lateral pathological areas (c) and only the volume of the tumours in the medial part of the brain (d) (mean ± SEM, unpaired two-sided t-test). Source Data are provided as a Source Data file for Supplementary Fig. 13a-d.

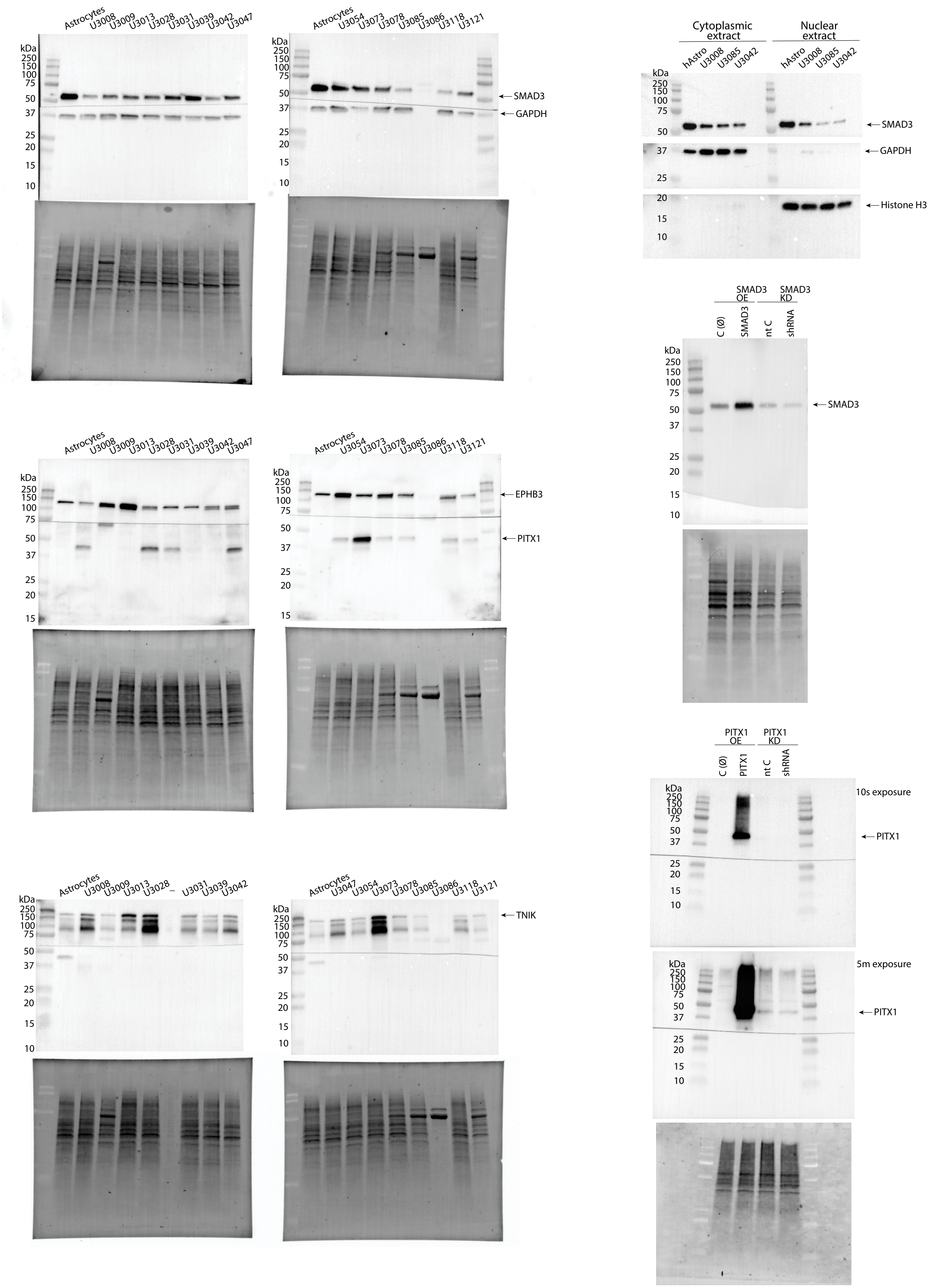

Supplementary Figure 14: Uncropped blots. Uncropped blots corresponding to the blots displayed in Supplementary Figs. 10a, c, e, g, i and 12g, h (also included in the Source Data file for Supplementary Fig. 14).
