# Supplementary material for: Rewiring of the promoter-enhancer interactome and regulatory landscape in glioblastoma orchestrates gene expression underlying neurogliomal synaptic communication
Source: Nat Commun. 2023 Oct 13;14:6446. doi: 10.1038/s41467-023-41919-x (PMC10576091; doi:10.1038/s41467-023-41919-x)
Supplement: Supplementary file 9 — Reporting Summary [file 41467_2023_41919_MOESM9_ESM.pdf]

nature portfolio

Corresponding author(s): Silvia Remeseiro

Last updated by author(s): Sep 20, 2023

Reporting Summary

Nature Portfolio wishes to improve the reproducibility of the work that we publish. This form provides structure for consistency and transparency in reporting. For further information on Nature Portfolio policies, see our Editorial Policies and the Editorial Policy Checklist.

Statistics

For all statistical analyses, confirm that the following items are present in the figure legend, table legend, main text, or Methods section.

n/a	Confirmed	
[ ]	[x]	The exact sample size (*n*) for each experimental group/condition, given as a discrete number and unit of measurement
[ ]	[x]	A statement on whether measurements were taken from distinct samples or whether the same sample was measured repeatedly
[ ]	[x]	The statistical test(s) used AND whether they are one- or two-sided<br>*Only common tests should be described solely by name; describe more complex techniques in the Methods section.*
[x]	[ ]	A description of all covariates tested
[ ]	[x]	A description of any assumptions or corrections, such as tests of normality and adjustment for multiple comparisons
[ ]	[x]	A full description of the statistical parameters including central tendency (e.g. means) or other basic estimates (e.g. regression coefficient) AND variation (e.g. standard deviation) or associated estimates of uncertainty (e.g. confidence intervals)
[ ]	[x]	For null hypothesis testing, the test statistic (e.g. *F*, *t*, *r*) with confidence intervals, effect sizes, degrees of freedom and *P* value noted<br>*Give P values as exact values whenever suitable.*
[x]	[ ]	For Bayesian analysis, information on the choice of priors and Markov chain Monte Carlo settings
[x]	[ ]	For hierarchical and complex designs, identification of the appropriate level for tests and full reporting of outcomes
[ ]	[x]	Estimates of effect sizes (e.g. Cohen's *d*, Pearson's *r*), indicating how they were calculated

Our web collection on statistics for biologists contains articles on many of the points above.

Software and code

Policy information about availability of computer code

Data collection	- TCGAbiolinks (2.18.0, 2.25.3) and RTCGAToolbox (2.20.0, 2.28.4) were used to retrieve RNA-seq data available at The Cancer Genome Atlas (TCGA, https://portal.gdc.cancer.gov/) from both human Glioblastoma (GB, n=156) and Low-Grade Glioma (LGG, n=511) samples alongside normal tissue samples.<br>- Next Generation Sequencing: NovaSeq 6000 System (Illumina).<br>- Incucyte Base Analysis Software was used to acquire and analyze live-cell imaging data obtained with the IncuCyte S3 Live-Cell Analysis instrument (Sartorius).<br>- Paravision 7.0 Software was use to collect MRI data on a Bruker MR scanner.<br>- LAS X software was used to collect immunofluorescence images with a Leica widefield Thunder microscope, whereas NIS-Elements (F 4.60.00) software was used to acquire those on the Nikon Eclipse E800 and a microscope.<br>-The Human Protein Atlas resource was used to retrieve protein expression and pathological information from glioma specimens.<br>- FACS sorting was performed on a BD FACSAria™ III Cell Sorter instrument using the BD FACSDiva software.
Data analysis	- Publicly available tools used in this study:<br>STAR (2.7.2b)<br>bowtie2 (2.4.1, 2.4.5)<br>samtools (1.12, 1.17)<br>bedtools (2.23.0, 2.25.0, 2.26.0, 2.27.1, 2.30.0)<br>deepTools (2.4.3, 2.5.1, 3.1.0, 3.3.2)<br>macs2 (2.2.6)<br>hichipper (0.7.3)

hicpro (v3.1.0)
fastqc (0.11.8, 0.11.9)
trimmomatic (0.39)
SEACR (v1.3)
picard (2.27.5)
java (sun_jdk1.8.0_151)

-Python (3.7.6) packages used in this study:
numpy (1.20.3)
pandas (1.3.3)
pybedtools (0.8.1)
sklearn (1.0)
seaborn (0.11.2)
matplotlib (3.4.3)

-Open source tools available via Bioconductor (3.11, 3.12, 3.13, 3.14, 3.15, 3.16), R environment (v.4.0.0-v.4.3.0)
DESeq2 (1.30.1, 13.38.3)
clusterProfiler (4.0.5, 4.6.2)
GenomicInteractions (1.26.0)
ChIPpeakAnno (3.26.4, 3.32.0)
ChIPseeker (1.28.3, 1.34.1)
ComplexHeatmap (2.6.2, 2.14.0)
cicrlize (0.4.15)
diffloops (1.20.0)
dplyr (1.1.3)
plyr (1.8.8)
reshape2 (1.4.4)
ggmisc (0.5.4-1)
ggpubr (0.6.0)
ggplot2 (3.4.3)
Rcircos (1.2.0)
rstatix (0.7.2)
EnsDb.Hsapiens.v86 (2.99.0)
BSgenome.Hsapiens.UCSC.hg38 (1.4.3, 1.44)

-HOMER (V4.119)
- MEMEsuite (v 5.5.1)
-ChromHMM 1.23
-GraphPad Prism 9
-Fiji (Image J v1)

For manuscripts utilizing custom algorithms or software that are central to the research but not yet described in published literature, software must be made available to editors and reviewers. We strongly encourage code deposition in a community repository (e.g. GitHub). See the Nature Portfolio guidelines for submitting code & software for further information.

Data

Policy information about availability of data

All manuscripts must include a data availability statement. This statement should provide the following information, where applicable:

- Accession codes, unique identifiers, or web links for publicly available datasets
- A description of any restrictions on data availability
- For clinical datasets or third party data, please ensure that the statement adheres to our policy

The RNA-seq, ChIP-seq, ATAC-seq, HiChIP and CUT&RUN datasets generated in this study have been deposited in GEO (Gene Expression Omnibus) under the accession number GSE217349, including the following SubSeries:

GSE217029 - ATAC - 15 GB and Astrocytes
GSE217031 - H3K27ac ChIP-seq - 15 GB and Astrocytes
GSE217035 - H3K27me3 ChIP-seq - 15 GB and Astrocytes
GSE217344 - H3K4me3 ChIP-seq - 15 GB and Astrocytes
GSE217346 - HiChIP - 15 GB and Astrocytes
GSE217348 - RNA-seq - 15 GB and Astrocytes
GSE234124 - ATAC - OPCs
GSE234125 - H3K27ac and H3K27me3 ChIP-seq - OPCs
GSE234126 - RNA-seq - OPCs
GSE234127 - CUT&RUN (SMAD3 and PITX1) - 13 GB, Astrocytes and OPCs

All sequencing data generated in this study has been mapped to the GRCh38/hg38 human genome.

The publicly available GB and LGG TCGA data was retrieved from The Cancer Genome Atlas (TCGA) data portal (https://portal.gdc.cancer.gov/).

Human research participants

Policy information about studies involving human research participants and Sex and Gender in Research.

Reporting on sex and gender	N/A
Population characteristics	N/A
Recruitment	N/A
Ethics oversight	N/A

Note that full information on the approval of the study protocol must also be provided in the manuscript.

Field-specific reporting

Please select the one below that is the best fit for your research. If you are not sure, read the appropriate sections before making your selection.

☒ Life sciences ☐ Behavioural & social sciences ☐ Ecological, evolutionary & environmental sciences

For a reference copy of the document with all sections, see nature.com/documents/nr-reporting-summary-flat.pdf

Life sciences study design

All studies must disclose on these points even when the disclosure is negative.

Sample size	15 patient-derived glioblastoma cell lines, one primary line of normal human astrocytes and oligodendrocyte progenitor cells (OPCs) were used in this study for RNA-seq, ChIP-seq, ATAC-seq, HiChIP and CUT&RUN. No statistical methods were used to predetermine sample size. The number of glioblastoma cell lines was chosen to represent all four GB subtypes (mesenchymal, classical, pro-neural and neural), keeping the proportions of their incidence in the human population and including 2-5 cell lines per GB subtype.
Data exclusions	All the RNA-seq, ATAC-seq, ChIP-seq, CUT&RUN and HiChIP data generated in the glioblastoma lines, the normal human astrocytes and the OPCs was included in this study, with the sole exception of the HiChIP in the U3047 line which was excluded from the analysis since it did not reach the standard quality criteria after sequencing and loop calling.
Replication	For each and all of the 15 glioblastoma lines, the normal human astrocytes and OPCs, we performed: a) RNA-seq in duplicates and sequenced ~50 million reads (PE150) per sample, b) ChIP-seq, ATAC-seq and HiChIP in single replicas and sequenced ~50, ~60 and ~100 million reads (PE150), respectively. For details on the number of reads per sample, please refer to Supplementary Tables S1-3. CUT&RUN for SMAD3 and PITX1 was performed in 13 glioblastoma lines, astrocytes and OPCs in single replicas and sequenced ~7 million reads (PE150).<br>Live-cell imaging experiments consist of 6-8 technical replicates per condition and time-point. These were performed at two different time-points in the induction protocol and using neurons generated in two independent reprogramming experiments.<br>In vivo experiments were performed in n=4-5 mice per group in longitudinal studies where MRI images were acquired weekly at 5 time-points between weeks 4 and 8 after tumour induction.<br>All the attempts at data replication were successful.
Randomization	Randomization is not relevant to most of our study since we apply omics approaches to each and all of the glioblastoma cell lines alongside normal astrocytes and OPCs as controls.<br>Randomization was performed in the in vivo studies, where mice were assigned randomly to the control and treatment groups i.e., vehicle-treated and SIS3-treated groups, respectively.
Blinding	The investigator performing live-cell imaging analysis was blinded to group allocation during data collection and image analysis.<br>For in vivo experiments, the investigator was not blinded to groups since the data was acquired and analyzed by the same person.

Reporting for specific materials, systems and methods

We require information from authors about some types of materials, experimental systems and methods used in many studies. Here, indicate whether each material, system or method listed is relevant to your study. If you are not sure if a list item applies to your research, read the appropriate section before selecting a response.

Materials & experimental systems

n/a	Involved in the study
	x Antibodies
	x Eukaryotic cell lines
x	Palaeontology and archaeology
	x Animals and other organisms
x	Clinical data
x	Dual use research of concern

Methods

n/a	Involved in the study
	x ChIP-seq
x	Flow cytometry
	x MRI-based neuroimaging

Antibodies

Antibodies used

Antibody Source ID Application Dilution Lot # Clonality Clone number
H3K27ac Abcam ab4729 ChIP-seq 4ug- Lot# GR3251520-1 Rabbit polyclonal
H3K27me3 Abcam ab192985 ChIP-seq 4ug - Lot# GR3204355-11 Rabbit monoclonal EPR18607
H3K4me3 Abcam ab8580 ChIP-seq 4ug - Lot# GR3362386-2 Rabbit polyclonal
H3K4me3 Abcam ab8580 HiChIP 4ug - Lot# GR3362386-2 Rabbit polyclonal
SMAD3 Abcam ab208182 CUT&RUN 1.2ug - Lot# GR3395943-9 Rabbit monoclonal EPR19686
PITX1 Santa Cruz sc-271435 CUT&RUN 4ug - Lot# KO121 Mouse monoclonal G-4
Beta III tubulin Abcam ab18207 IF 1:1000 - Lot# GR3430488-1 Rabbit polyclonal
SMAD3 Abcam ab208182 IF 1:500 - Lot# GR3395943-9 Rabbit monoclonal EPR19686
PITX1 Santa Cruz sc-271435 IF 1:200 - Lot# KO121 Mouse monoclonal G-4
TNIK Abcam ab224252 IF 2ug/ml - Lot# 1016345-1 Rabbit polyclonal
EPHB3 Abcam ab133742 IF 1:150 - Lot# 1002215-1 Rabbit monoclonal EPR8280
KCNE4 Abcam ab254642 IF 2ug/ml - Lot# 1039028-1 Rabbit polyclonal
Goat anti-rabbit AF594 Invitrogen A32740 IF 1:500 - Lot# WK333741 Polyclonal
Goat anti-mouse AF488 Invitrogen A32723TR IF 1:500 - Lot# VE306232 Polyclonal
SMAD3 Abcam ab208182 Western-Blot 1:1000 - Lot# GR3395943-9 Rabbit polyclonal
PITX1 Thermo Fisher A300-577A-T Western-Blot 1:1000 - Lot# 2 Rabbit polyclonal
TNIK Abcam ab224252 Western-Blot 1:1000 - Lot# 1016345-1 Rabbit polyclonal
EPHB3 Abcam ab133742 Western-Blot 1:1000 - Lot# 1002215-1 Rabbit monoclonal EPR8280
GAPDH Cell Signaling #14C10 Western-Blot 1:1000 - Lot# 14 Rabbit polyclonal
Histone H3 Abcam ab176842 Western-Blot 1:8000 - Lot# 1000615-6 Rabbit monoclonal 14C10
Peroxidase AffiniPure Goat Anti-Rabbit IgG (H+L) Jackson ImmunoResearch 111-035-003 Western-Blot 1:10000 - Lot# 104122 Monoclonal EPR16987

Validation

H3K27ac (Abcam ab4729, ChIP-grade antibody): https://www.abcam.com/histone-h3-acetyl-k27-antibody-chip-grade-ab4729.html
H3K27me3 (Abcam ab192985, ChIP-grade antibody): https://www.abcam.com/histone-h3-tri-methyl-k27-antibody-epr18607-chip-grade-ab192985.html
H3K4me3 (Abcam ab8580, ChIP-grade antibody): https://www.abcam.com/histone-h3-tri-methyl-k4-antibody-chip-grade-ab8580.html
SMAD3 (Abcam ab208182, ChIP-grade antibody, KO validated): https://www.abcam.com/products/primary-antibodies/smad3-antibody-epr19686-chip-grade-ab208182.html
Beta III tubulin (Abcam ab18207, KO validated): https://www.abcam.com/beta-iii-tubulin-antibody-neuronal-marker-ab18207.html
TNIK Abcam ab224252 (Abpromise guarantee for use in tested applications: IF): https://www.abcam.com/products/primary-antibodies/tnik-antibody-ab224252.html
EPHB3 Abcam ab133742 (Abpromise guarantee for use in tested applications: IF and WB): https://www.abcam.com/products/primary-antibodies/eph-receptor-b3-antibody-epr8280-ab133742.html
KCNE4 Abcam ab254642 (Abpromise guarantee for use in tested applications: IF and WB): https://www.abcam.com/products/primary-antibodies/kcne4-antibody-ab254642.html
SMAD3 and PITX1 antibodies were also validated in our own knockdown and overexpression experiments.

Eukaryotic cell lines

Policy information about cell lines and Sex and Gender in Research

Cell line source(s)

- The following human glioblastoma cell lines (n=15) were obtained through the Human Glioblastoma Cell Culture (HGCC) resource (Uppsala University, Sweden, https://www.hgcc.se/): U3008, U3009, U3013, U3028, U3031, U3039, U3042, U3047, U3054, U3073, U3078, U3085, U3086, U3118, U3121. Of those, n=5 classical, n=5 mesenchymal, n=3 pro-neural and n=2 neural subtype.
- Normal Human Astrocytes (NHAs): Lonza (CC-2565)
- Human iPSC-Derived Glutamatergic Neurons (ioGlutamatergic Neurons): Abcam (ab259259)
- OPCs: iNeuTM human oligodendrocyte progenitor cells (OPCs): Creative Biolabs NeuroS (NCL-2103-P49)
- U251 (glioblastoma cell line): Sigma (09063001-1VL)

Authentication

- The 15 glioblastoma lines were obtained directly from HGCC, a resource that provides newly established and characterized

GB cell lines from GB patient surgical samples. The resource characterizes the lines in terms of gene expression, copy number and subtype classification, among others.
- Normal Human Astrocytes (NHAs): certificate of analysis provided by Lonza for each lot.
- iPSC-derived Glutamatergic Neurons: characterized by the expression of glutamate transporter genes VGLUT1 and VGLUT2 and markers FOXG1 and TBR1 (RNA-seq, at Abcam).
- OPCs (oligodendrocyte progenitor cells): characterized by standard morphological examination and immunocytochemical methods for known marker proteins (such as O4, PDGFalphaR, NG2 and CNPase) at Creative Biolabs NeuroS.
- U251 (glioblastoma cell line): authenticated by Sigma by STR-PCR profiling.

Mycoplasma contamination

All cell lines tested negative for mycoplasma using the MycoAlert PLUS detection kit (Lonza, LT07-703).

Commonly misidentified lines
(See ICLAC register)

No commonly misidentified cell lines were used in the study.

Animals and other research organisms

Policy information about studies involving animals; ARRIVE guidelines recommended for reporting animal research, and Sex and Gender in Research

Laboratory animals

- Mus musculus, NSG mouse strain (NOD.Cg-Prkdcscid Il2rgtm1Wjl/SzJ).
- Mice were housed under 12:12 h light:dark cycle conditions in temperature- and humidity-controlled rooms (22 °C and 50% humidity).
- Tumour formation was induced by intracranial injection in neonatal mice (P1, P2) and tumour progression was monitored until the experiment end-point when the mice were 8 weeks old.

Wild animals

The study did not involve wild animals.

Reporting on sex

Mice of both sexes were used in this study. Tumour formation was induced by orthotopic transplantation of neonatal mice, an early time-point at which sex is not assessed, and all pups were injected to have littermate controls and representation from both sexes.

Field-collected samples

The study did not involve field-collected samples.

Ethics oversight

All experiments with laboratory animals were performed in compliance with national and institutional laws, and according to protocols approved by the Regional Ethics Committee at the Court of Appeal of Northern Norrland (ethical permit ID A29-2019 and A3-2023).

Note that full information on the approval of the study protocol must also be provided in the manuscript.

ChIP-seq

Data deposition

☒ Confirm that both raw and final processed data have been deposited in a public database such as GEO.

☒ Confirm that you have deposited or provided access to graph files (e.g. BED files) for the called peaks.

Data access links
May remain private before publication.

https://www.ncbi.nlm.nih.gov/geo/query/acc.cgi?acc=GSE217349
GEO accession number GSE217349
ChIP-seq subseries GSE217031 (H3K27ac), GSE217035 (H3K27me3), GSE217344 (H3K4me3) for GB lines and astrocytes; and GSE234125 (H3K27ac, H3K27me3) for OPCs.

Files in database submission

GSM6703836 astrocytes, cells, control (H3K27Ac.ChIPseq)
GSM6703837 U3008, cells, GBM (H3K27Ac.ChIPseq)
GSM6703838 U3009, cells, GBM (H3K27Ac.ChIPseq)
GSM6703839 U3013, cells, GBM (H3K27Ac.ChIPseq)
GSM6703840 U3028, cells, GBM (H3K27Ac.ChIPseq)
GSM6703841 U3031, cells, GBM (H3K27Ac.ChIPseq)
GSM6703842 U3039, cells, GBM (H3K27Ac.ChIPseq)
GSM6703843 U3042, cells, GBM (H3K27Ac.ChIPseq)
GSM6703844 U3047, cells, GBM (H3K27Ac.ChIPseq)
GSM6703845 U3054, cells, GBM (H3K27Ac.ChIPseq)
GSM6703846 U3073, cells, GBM (H3K27Ac.ChIPseq)
GSM6703847 U3078, cells, GBM (H3K27Ac.ChIPseq)
GSM6703848 U3085, cells, GBM (H3K27Ac.ChIPseq)
GSM6703849 U3086, cells, GBM (H3K27Ac.ChIPseq)
GSM6703850 U3118, cells, GBM (H3K27Ac.ChIPseq)
GSM6703851 U3121, cells, GBM (H3K27Ac.ChIPseq)
GSM6703852 astrocytes, cells, control (input for H3K27Ac)
GSM6703853 classical subtypes, cells, GBM (input for H3K27Ac)
GSM6703854 mesenchymal subtypes, cells, GBM (input for H3K27Ac)

GSM6703855 neural and proneural subtypes, cells, GBM (input for H3K27Ac)
GSM6703979 astrocytes, cells, control (H3K27Me3.ChIPseq)
GSM6703980 U3008, cells, GBM (H3K27Me3.ChIPseq)
GSM6703981 U3009, cells, GBM (H3K27Me3.ChIPseq)
GSM6703982 U3013, cells, GBM (H3K27Me3.ChIPseq)
GSM6703983 U3028, cells, GBM (H3K27Me3.ChIPseq)
GSM6703984 U3031, cells, GBM (H3K27Me3.ChIPseq)
GSM6703985 U3039, cells, GBM (H3K27Me3.ChIPseq)
GSM6703986 U3042, cells, GBM (H3K27Me3.ChIPseq)
GSM6703987 U3047, cells, GBM (H3K27Me3.ChIPseq)
GSM6703988 U3054, cells, GBM (H3K27Me3.ChIPseq)
GSM6703989 U3073, cells, GBM (H3K27Me3.ChIPseq)
GSM6703990 U3078, cells, GBM (H3K27Me3.ChIPseq)
GSM6703991 U3085, cells, GBM (H3K27Me3.ChIPseq)
GSM6703992 U3086, cells, GBM (H3K27Me3.ChIPseq)
GSM6703993 U3118, cells, GBM (H3K27Me3.ChIPseq)
GSM6703994 U3121, cells, GBM (H3K27Me3.ChIPseq)
GSM6703995 astrocytes, cells, control (input for H3K27Me3)
GSM6703996 classical subtypes, cells, GBM (input for H3K27Me3)
GSM6703997 mesenchymal subtypes, cells, GBM (input for H3K27Me3)
GSM6703998 neural and proneural subtypes, cells, GBM (input for H3K27Me3)
GSM6715172 hAstro, cells, control (H3K4me3.ChIPseq)
GSM6715173 U3008, cells, GBM (H3K4me3.ChIPseq)
GSM6715174 U3009, cells, GBM (H3K4me3.ChIPseq)
GSM6715175 U3013, cells, GBM (H3K4me3.ChIPseq)
GSM6715176 U3028, cells, GBM (H3K4me3.ChIPseq)
GSM6715177 U3031, cells, GBM (H3K4me3.ChIPseq)
GSM6715178 U3039, cells, GBM (H3K4me3.ChIPseq)
GSM6715179 U3042, cells, GBM (H3K4me3.ChIPseq)
GSM6715180 U3047, cells, GBM (H3K4me3.ChIPseq)
GSM6715181 U3054, cells, GBM (H3K4me3.ChIPseq)
GSM6715182 U3073, cells, GBM (H3K4me3.ChIPseq)
GSM6715183 U3078, cells, GBM (H3K4me3.ChIPseq)
GSM6715184 U3085, cells, GBM (H3K4me3.ChIPseq)
GSM6715185 U3086, cells, GBM (H3K4me3.ChIPseq)
GSM6715186 U3118, cells, GBM (H3K4me3.ChIPseq)
GSM6715187 U3121, cells, GBM (H3K4me3.ChIPseq)
GSM6715188 hAstro, cells, control (input for H3K4me3)
GSM6715189 classical subtypes, cells, GBM (input for H3K4me3)
GSM6715190 mesenchymal subtypes, cells, GBM (input for H3K4me3)
GSM6715191 neural and proneural subtypes, cells, GBM (input for H3K4me3)
GSM7446474 OPC, cells, control (H3K27Ac ChIP)
GSM7446475 OPC, cells, control (H3K27Me3 ChIP)
GSM7446476 OPC, cells, control (input for H3K27Ac and H3K27Me3 ChIP)

Genome browser session
(e.g. UCSC)

Provide a link to an anonymized genome browser session for "Initial submission" and "Revised version" documents only, to enable peer review. Write "no longer applicable" for "Final submission" documents.

Methodology

Replicates

ChIP-seq was performed in 15 glioblastoma cell lines and normal human astrocytes in single replicas with antibodies against H3K27ac, H3K27me3 and H3K4me3. For OPCs (oligodendrocyte progenitor cells), ChIP-seq was performed for H3K27ac and K3K27me3 in single replicates.

Sequencing depth

The ChIP-seq libraries were sequenced on a NovaSeq 6000 Sequencing System (Illumina) obtaining ~54 million 150PE reads per library. Details about the total number of reads, percentage of uniquely mapped reads and number of peaks called per sample can be found in Supplementary Table S3.

Antibodies

Antibody Source ID Application Dilution
H3K27ac Abcam ab4729 ChIP-seq 4ug
H3K27me3 Abcam ab192985 ChIP-seq 4ug
H3K4me3 Abcam ab8580 ChIP-seq 4ug

Peak calling parameters

Peak calling was performed over input using MACS2 (options: --broad -g hs -B -q 0.05)

Data quality

Fastq files were quality-checked with FastQC (https:// www.bioinformatics.babraham. ac.uk/projects/fastqc/) and significant enrichment over input was determined by peak calling using MACS2 at q 0.05.

Software

-Mapping (GRCh38/hg38): bowtie2 (2.4.1)
-Peak calling: macs2 (2.2.6)

-samtools (1.12)
-bedtools (2.23.0, 2.25.0, 2.26.0, 2.27.1)
-deepTools (2.4.3, 2.5.1, 3.1.0, 3.3.2)
-wiggletools (1.2, EMBL-EBI)
-fastqc (0.11.8)

Magnetic resonance imaging

Experimental design

Design type	n/a
Design specifications	n/a
Behavioral performance measures	n/a

Acquisition

Imaging type(s)	Structural
Field strength	9.4 Tesla
Sequence & imaging parameters	The imaging was performed in a Bruker MR scanner using a T2-weighted TurboRARE sequence; parameters: TR = 3.6 s; TE = 37 ms; RARE factor = 8; matrix size = 128 x 96; field of view = 12.8 x 9.6 mm; slice thickness = 0.2 mm, slices = 30; duration of acquisition=27 min22 sec.
Area of acquisition	whole brain scan (olfactory bulbs and cerebellum were not scanned)

Diffusion MRI ☐ Used ☒ Not used

Preprocessing

Preprocessing software	n/a
Normalization	n/a
Normalization template	n/a
Noise and artifact removal	n/a
Volume censoring	n/a

Statistical modeling & inference

Model type and settings	n/a
Effect(s) tested	n/a

Specify type of analysis: ☐ Whole brain ☐ ROI-based ☐ Both

Statistic type for inference (See Eklund et al. 2016)	n/a
Correction	n/a

Models & analysis

n/a	Involved in the study
☒	☐ Functional and/or effective connectivity
☒	☐ Graph analysis
☒	☐ Multivariate modeling or predictive analysis
